# Study on the performance of thin-film VCSELs on composite metal substrate

**DOI:** 10.1007/s12200-023-00086-z

**Published:** 2023-11-08

**Authors:** William Anderson Lee Sanchez, Shreekant Sinha, Po-Yu Wang, Ray-Hua Horng

**Affiliations:** Institute of Electronics, Yang Ming Chiao Tung University, Hsinchu, 30010 China

**Keywords:** Thin film, VCSELs, GaAs substrate, Composite metal, CIC substrate, Twice-bonding transfer, Electrical properties, Heat dissipation

## Abstract

Thin film p-side up vertical-cavity surface-emitting lasers (VCSELs) with 940 nm wavelength on a composite metal (Copper/Invar/Copper; CIC) substrate has been demonstrated by twice-bonding transfer and substrate removing techniques. The CIC substrate is a sandwich structure with a 10 µm thick Copper (Cu) layer/30 µm thick Invar layer/10 µm thick Cu layer. The Invar layer was composed of Iron (Fe) and Nickel (Ni) with a proportion of 70:30. The thermal expansion coefficient of the composite CIC metal can match that of the GaAs substrate. It results that the VCSEL layers can be successfully transferred to CIC metal substrate without cracking. At 1 mA current, the top-emitting VCSEL/GaAs and thin-film VCSEL/CIC had a voltage of 1.39 and 1.37 V, respectively. The optical output powers of VCSEL/GaAs and VCSEL/CIC were 21.91 and 24.40 mW, respectively. The 50 µm thick CIC substrate can play a good heat dissipation function, which results in improving the electrical and optical characteristics of thin film VCSELs/CIC. The VCSEL/CIC exhibited a superior thermal management capability as compared with VCSEL/GaAs. The obtained data suggested that VCSELs on a composite metal substrate not only affected significantly the characteristics of thin film VCSEL, but also improved considerably the device thermal performance.

**Graphical Abstract:**

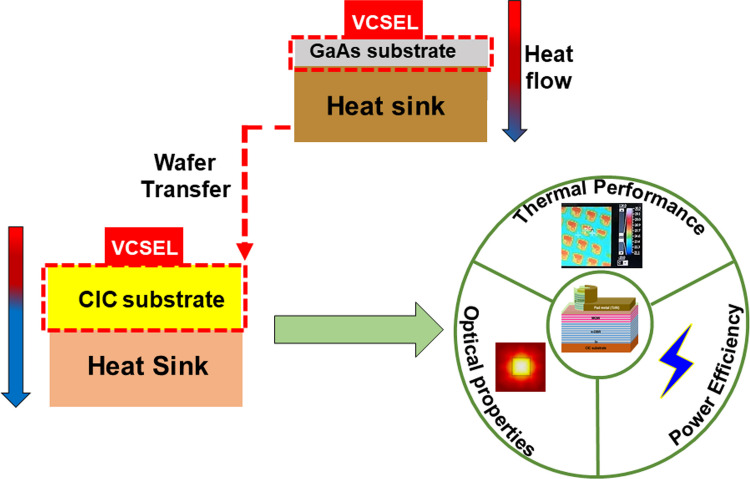

## Introduction

Demand for vertical-cavity surface-emitting lasers (VCSELs) has grown rapidly as a result of the cutting-edge applications based on VCSELs. It can be used in diverse consumer applications, including sensors, laser printers, and laser mice, in addition to serving as the light source in optical interconnect (OI) networks using multimode optical fibers [1–6]. VCSELs are a remarkable light source that are highly-preferred for use in optical data links due to their high modulation speed and low-power consumption at low threshold current, as well as a high circular optical beam quality that facilitates efficient optical coupling with other systems. These characteristics are in response to the requirement for high-speed data communication networks [7–10].

Typically, the epilayer structures for 850–940 nm VCSELs are grown on GaAs substrates. However, the theoretical thermal conductivity of GaAs substrates is only about 55 W/(m·K) [11–13], which could limit the VCSEL to be operated under high power. In corresponding, Copper-Invar-Copper (CIC) substrate has a high theoretical thermal conductivity of approximately 90–170 W/(m·K) [14–16]. Different studies have reported that thermal conductivity of this metal composite depends enormously on the Copper-Invar proportion [17, 18]. Obviously, the thermal conductivity of GaAs substrate is lower than that of CIC substrate. The optics transceiver modules (VCSELs with photodiodes) must be packaged as close to the electronic integrated circuits (ICs), which also generate a significant amount of heat during high-speed operations, making sustained high-speed performance of VCSELs under high-temperature operations a significant challenge [1, 19–21].

High-power VCSELs can only be secured to a certain limit, however, because of thermal resistance caused on by the greatly elevated temperature around the active layers at higher injection current [22–24], the device properties including the threshold current, voltage, output power, and emission spectrum are adversely affected [22, 25]. In addition, the self-heating impact shortens the operational lifetime of VCSEL, as a rise in temperature is able to ease the kinetic mobility and the development of dislocations, which in turn causes device aging and instability in the VCSEL reliability [22, 26, 27]. Many attempts have been made to transfer thin-film VCSELs onto heatsinks with the removal of the GaAs substrate in order to overcome this problem, which has improved the thermal properties of VCSELs [22, 28–33]. In general, Si substrate was used to replace GaAs and can play a good heat conductivity. In this work, CIC not only play well thermal conductivity, but also contribute to simple processing due to thin CIC and metal substrate [34].

The CIC substrate has already been proposed and fabricated based on the aforementioned studies [34–37]. The CIC substrate has a top and bottom Copper (Cu) layer with 10 μm thickness and a middle 30 μm thick Invar layer. The Invar layer was composed of Iron (Fe) and nickel (Ni) with a proportion of 70:30. The coefficient of thermal expansion (CTE) of the composite CIC metal can match that of the GaAs substrate. It results that the VCSEL layers can be successfully transferred to CIC metal substrate without cracking. Additionally, the CIC thickness is just 50 μm, which could result in high heat dissipation because thermal resistance is correlated with substrate thickness. This study effectively transferred a VCSEL epilayer from a GaAs substrate to the CIC substrate and the properties of VCSEL/GaAs and VCSEL/CIC were compared.

## Experimental section

### Device fabrication

**Fig. 1:**
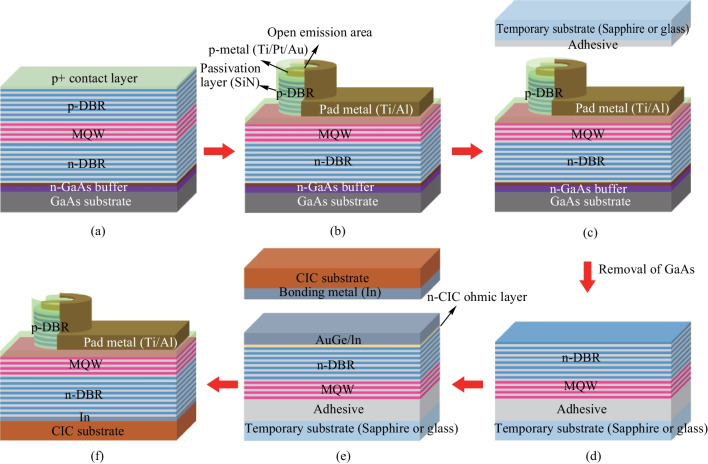
VCSEL device transfer procedure onto CIC substrate by double wafer bonding techniques and transfer technologies

VCSEL epitaxial structure was grown by metal–organic chemical vapor deposition (MOCVD), which consisted of a 625 μm thick GaAs substrate, an GaInP etching stop layer with 200 nm thickness, a n^+^-doped GaAs contact layer, 5.8 μm thick n-type diffraction Bragg reflectors (DBRs), a 350 nm thick active layer, a 25 nm thick oxide layer, a 3.2 μm thick p-type DBRs, a 10 nm thick p^+^-doped GaAs contact layer, as illustrated in Fig. 1a. First, Au/Pt/Ti (100/50/15 nm) metals were deposited on the highly p^+^-doped GaAs contact layer as p-electrode. After that, the SiN layer with 0.5 μm thickness was grown using a plasma-enhanced chemical vapor deposition (PECVD) system as a hard mask. Then, the SiN layer and epilayers were etched to exposure the oxide layer. Selective oxidation of Al_*x*_Ga_1−*x*_As with high Al concentrations was used to define the 8 μm oxide-confined aperture size of each VCSEL (It means the diameter of the active area was 8 μm) and the oxidation process was carried out at 430 °C for 30 min. After, the 1 μm thick SiN later was deposited for passivation, then the emission area and p-metal electrode were exposed. Subsequently, the outside pad metal (Ti/Al) was deposited for connecting to the p-metal electrodes, shown in Fig. 1b. After finished the p-side up VCSEL process, the epiwafer was divided into two parts. One part was deposited the backside metal Au/Ge on GaAs substrate. The other part was transferred to CIC substrate and described as following.

In order to obtain the thin film VCSEL/CIC sample, the emission side of finished VCSEL device was temporarily bonded on sapphire substrate by adhesive layer, shown in Fig. 1c. The GaAs substrate for the VCSEL epilayer growth was removed to etching stop at GaInP layer using a wet etchant of NH_4_OH:H_2_O_2_, shown in Fig. 1d. Noted that the GaInP with 200 nm thickness was used to be the etching stop layer. There existed a high etching selectivity of NH_4_OH:H_2_O_2_ to etching GaAs substrate and stop at the GaInP layer. After the GaAs substrate removing, the epilayer was etched to stop at the GaInP, which presented the bright red color, due to the high chemical selectivity. Further removing the GaInP, the color of epilayer will change to dark red color due to n^+^-GaAs absorbing the visible light. The changed color can be used to judge whether etching stop layer etched away or not and also ensure the contact metal being deposited on n^+^-GaAs. This technology has well applied to transfer the red AlGaInP/GaAs to n-side up AlGaInP/mirror/Si substrate [37]. After etching away the etching stop layer, AuGe and indium metals were deposited for contact with n^+^-GaAs and bonding with CIC [33]. Acetone (ACE) and diluted hydrochloric acid (HCl) were used to clean the CIC substrate in order to achieve strong adhesion on the surface. After cleaning, the bonding metal indium with a 2 μm thickness was deposited. The In/AuGe/VCSEL device/adhesive on sapphire or glass and In/CIC substrate was bonded at 300 °C under the pressure of 48 N/cm^2^ for 3600 s [38], as shown in Fig. 1e. Following that, the entire VCSEL device was successfully transferred onto the composite metal substrate with 50 µm after the removal of the temporary substrate and adhesive, as illustrated in Fig. 1f. It was worthy to mention that because the CIC is metal and was directly contact to n-metal by In bonding metal, the CIC can be regarded n Ohmic contact layer. Note that the GaAs substrate cannot be thinned to 50 µm thickness due to its brittle property. Nevertheless, the GaAs substrate of the other samples VCSEL on GaAs substrate was thinned to 150 µm for comparison.

### Characterizations

This work involved the fabrication of VCSELs on GaAs substrate with 150-µm thickness and VCSELs on CIC substrate with 50-µm thickness using twice wafer bonding, epilayer transferring, and thermal oxidation technologies. A semiconductor parameter analyzer (Agilent 4155B) was employed to measure the voltage-current characteristics of the VCSELs with CIC and GaAs substrates. Output power and wavelength properties were obtained using an integrated sphere detector (CAS 140B, Instrument Systems). Thermal properties of the substrates were measured utilizing a thermal constant analyzer (TPS 2500S, Hot Disk, Sweden) at room temperature and at increased temperatures. The linear coefficient of thermal expansion of the samples was determined employing a thermal mechanical analyzer (TMA Q400, TA instruments, USA), the specimens were heated in an analyzer furnace under nitrogen atmosphere at a ramp rate of 5 °C/min from room temperature to 130 °C. Prior to packaging, infrared thermography equipment (Advanced Thermo TVS-500EX, Avio, Japan) was used to measure the surface temperature distribution and thermal transfer ability of the VCSEL devices on the GaAs and CIC substrates [39].

## Results and discussion

### Thermal properties of GaAs and CIC substrates

**Fig. 2:**
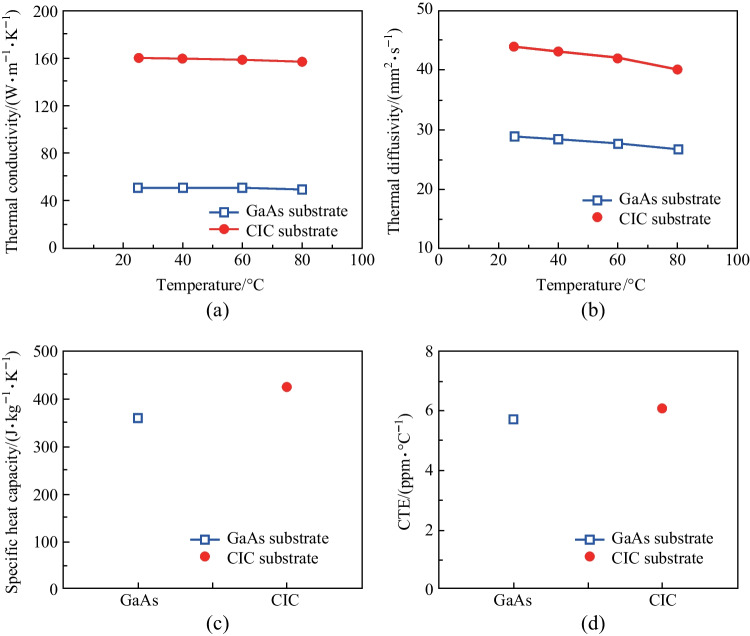
Thermal properties of VCSEL/GaAs and VCSEL/CIC substrates. **a** Thermal conductivity. **b** Thermal diffusivity. **c** Specific heat capacity. **d** Linear coefficient of thermal expansion

First, it is important to evaluate the thermal properties of the CIC substrate as compared with those of GaAs substrate as shown in Fig. 2. As observed in Fig. 2a, CIC substrate exhibits a thermal conductivity of 160 W/(m·K) at room temperature, which is about 3.1 times of that (51.1 W/(m·K)) of the GaAs substrate. This result is ascribed to the inherent characteristics of the Cu material [40, 41], one of the individual components of the composite metal substrate, which provides high thermal conductivity on the CIC substrate. In addition, thermal conductivity tends to decrease with increased temperature on metals, for this reason the thermal conductivity of both substrates was measured at 80 °C, the CIC substrate presented a thermal conductivity of (157.1 W/(m·K)), while GaAs substrate showed a thermal conductivity of (49.49 W/(m·K)). There existed 1.8% and 3.1% reducing thermal conductivity for the CIC and GaAs substrates, respectively. Even with the decrease of thermal conductivity due to the increase of ambient temperature, the thermal conductivity of CIC substrate is still superior than that of GaAs substrate, which indicates that substrates made of CIC materials can effectively play thermal dissipation at intense temperature during device operation. Figure 2b showed the thermal diffusivity as function of temperature for the GaAs and CIC substrates. The thermal diffusivity decreased as the temperature increased for both substrates. The thermal diffusivity of CIC substrate was higher than that of GaAs substrate. Figure 2c presented the specific heat capacity of GaAs and CIC substrates. The specific heat capacity of CIC and GaAs substrate was 450 and 360 J/(kg·K), respectively. The specific heat capacity is defined as the quality of heat absorbed or dissipated per unit mass of the material as its temperature increases or decreases. Obviously, CIC can absorb and dissipate the heat much quicker than GaAs does. Furthermore, one of the crucial elements for the VCSEL wafer bonding and epilayer transferring applications was the CTE of the CIC substrate. As illustrated in Fig. 2d, the CTE of GaAs substrate was 5.71 ppm/°C and the CTE of CIC substrate was 6.1 ppm/°C. Although the Cu material possesses a little bigger thermal expansion in relation to the GaAs substrate, both the CTE of GaAs and CIC materials were almost matched, which resulted from the Invar material intrinsic properties of the composite metal substrate. This contributed to the VCSEL epilayer transferring to CIC substrate without cracking.

### Optoelectronic characteristics of VCSEL/GaAs and VCSEL/CIC devices

**Fig. 3:**
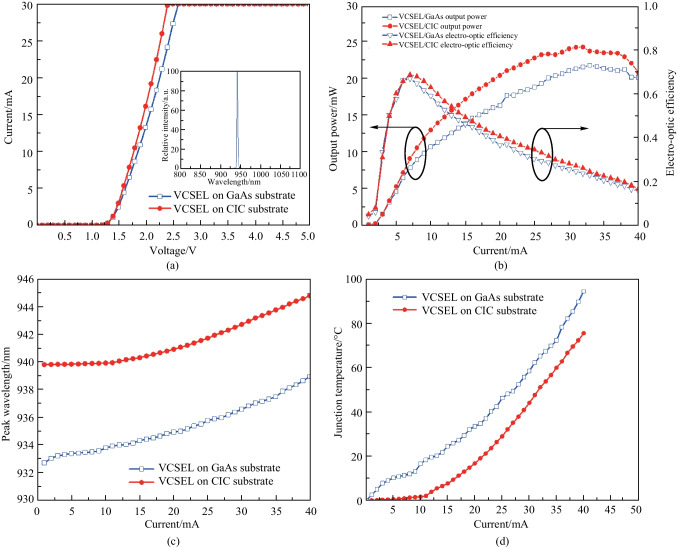
Electrical characteristics of VCSEL devices. **a** *I–V* curve and spectra of VCSEL/GaAs and VCSEL/CIC. **b** Output power and electro-optic efficiency of VCSEL/GaAs and VCSEL/CIC versus current. **c** Wavelength-current characteristics of VCSEL/GaAs and VCSEL/CIC versus current. **d** Junction temperature of VCSEL/GaAs and VCSEL/CIC versus current

In this study, the VCSEL epilayer can be successfully transferred to CIC substrate by twice wafer bonding and GaAs substrate removing. The most significant concern was VCSEL performance when the epilayer was transferred from the GaAs substrate to the CIC substrate, therefore, several essential electrical properties for the application were analyzed, as presented in Fig. 3. Figure 3a compares the characteristics of current as a function of voltage for both the VCSEL/GaAs and VCSEL/CIC. It was found that the cut-in voltage was almost the same (about 1.25 V) for both VCSELs. After turning on the VCSELs, the dynamic resistances (@ 10 mA) for VCSEL on GaAs and CIC substrates were 175 and 190 Ω, respectively. As applying an injection current of 1 mA, the VCSEL/CIC presented a forward voltage of (1.37 V), which is slightly lower as compared with that of the VCSEL/GaAs (1.39 V). Obviously, the VCSEL/CIC device presented improved electrical characteristics. Based on the above results, VCSEL devices on a CIC substrate possessed superior electrical characteristics owing to the inherent properties of the composite metal substrate, such as, high thermal conductivity and thinner thickness as compared with those of the GaAs substrate. The typical spectrum of VCSEL on CIC injected by 30 mA was shown in the inset of Fig. 3a. Obviously, the spectrum of VCSEL/CIC presented a narrow distribution, which proved the successful fabrication of a VCSEL device.

The output power and electro-optic efficiency for the optoelectronic performance of the VCSEL/GaAs and VCSEL/CIC were measured as a function of increase in current, and displayed in Fig. 3b. There existed two regions in the output power-current curves. One was linear region and the other was nonlinear region. It was found that linear region of VCSEL with CIC substrate can be operated at higher injection current (9.5 mA) as compared with that of VCSEL with GaAs substrate (7.5 mA). Moreover, the output power of VCSEL/GaAs and VCSEL/CIC exhibited a saturation at 33 mA, then dropped after 33 mA. The VCSEL/GaAs and VCSEL/CIC devices have maximum output powers of around 21.91 and 24.40 mW, respectively. It contributed to 11% output power increasing for the VCSEL/CIC than that of VCSEL/GaAs. Noted that the output powers were almost the same for both devices as the injection current was below 4 mA. It indicated the self-heating occurred after 4 mA injection current. Furthermore, it was found that the electro-optic efficiency of VCSEL/CIC was superior than that of VCSEL/GaAs, for example, the peak electro-optic efficiency (68%, @ 8 mA) of VCSEL/CIC was slightly higher than (67%, @ 7 mA) that of VCSEL/GaAs.

The wavelength characteristics of both the VCSEL/GaAs and VCSEL/CIC versus a function of the forward current were shown in Fig. 3c. The VCSEL/GaAs wavelength red-shifted approximately from 932.74 to 938.98 nm, while the VCSEL/CIC wavelength red-shifted approximately from 939.84 to 944.83 nm as the injection current was 1 to 40 mA. Its wavelength variations are 0.16 and 0.12 nm/mA, respectively. Because the CIC can provide a good thermal dissipation, it resulted in the VCSEL/CIC presented the less wavelength variation. It was worthy to mention that the initial wavelength of VCSEL/CIC was about 940 nm, which was longer than that of VCSEL/GaAs. It could be resulted from the stress released after the GaAs substrate removing.

The results indicate that VCSEL devices on CIC composite metal substrates significantly improve the thermally driven spectral detuning throughout the gain region and cavity [42–46]. According to the literature, the gain peak wavelength possesses a strong dependence on temperature mostly because of bandgap shrinkage with increase of internal operating temperature. This effect leads to the gain peak and operating wavelength shifting at various rates as a function of temperature, which is known as cavity resonance detuning. Since the VCSEL devices are designed for operation at a specific wavelength, temperatures variations might lead to a reduction of the maximum operating power and hasten the rollover effect. Moreover, the power dissipated in the laser is the main driving force of the unsought internal temperature accumulation, causing reflectivity shifts in DBRs. The variation in reflectivity generates a disturbance on the ideal device performance conditions, thus, more power begins to be transfer into heat, instead of being absorbed in the active region. As a result, decreasing the temperature created by the internal self-heating of the VCSEL is vital for the device application.

As noticed from the previous discussion, since the wavelength variation is correlated with the increase of the internal temperature during operation of the device, it is essential to evaluate the junction temperature of the VCSEL/GaAs and VCSEL/CIC. According to the literature [47–50], there are several methods to obtain directly or indirectly the junction temperature of photonic devices. Figure 3d exhibits the junction temperature as a function of injection current for both the VCSEL/GaAs and VCSEL/CIC devices. Since the wavelength variation of both types of VCSEL devices as a function of increased current during operation was measured experimentally, as presented in Fig. 3c, the junction temperature *T*_j_ dependence of the wavelength variation can be described by the equation below:

$${T}_{\mathrm{j}}={T}_{0}+\frac{({\lambda }_{1}-{\lambda }_{0})}{{R}_{\lambda }},$$

where *T*_0_ is the reference atmospheric temperature, *λ*_1_ is the peak wavelength of the VCSEL at the reference current for the test condition, *λ*_0_ is the reference peak wavelength at the reference current and temperature and *R*_*λ*_ equals the VCSEL wavelength temperature coefficient, which is a well-known characterized physical value, determined by the VCSEL lasing wavelength and it is mainly independent of the VCSEL aperture area and epitaxial design. For VCSELs operating at a wavelength of 940 nm, *R*_*λ*_ is 0.066 nm/°C. It was found that the VCSEL/GaAs and VCSEL/CIC exhibited junction temperatures of approximately 94.55 °C and 75.61 °C under 40-mA injection current, respectively. Furthermore, the junction temperature rate of the VCSEL/CIC by 1.93 °C/mA is lower as compared with that of the VCSEL/GaAs by 2.36 °C/mA. It implies that the heat dissipation of the VCSEL/CIC is better than that of the VCSEL/GaAs.

From the above discussion, it was determined that the VCSEL/CIC has superior laser characteristics, owing to the outstanding thermal properties of the CIC metal composite material, meaning that the CIC substrate is able to successfully prevent the laser device from a severe heat accumulation due to self-heating, which leads to a smaller wavelength shift as compared with VCSEL/GaAs, obtaining an improved device performance, making the VCSEL/CIC a quite promising photonic devices with high-power that are efficient under temperature variations.

### Thermal performance of VCSEL/GaAs and VCSEL/CIC devices at steady state

Prior to packaging, the temperature distribution across the surfaces of the VCSEL/GaAs and VCSEL/CIC substrates were evaluated. First, all specimens were maintained under room temperature for 30 min to ensure the experimental system has reached the condition of thermal equilibrium. Subsequently, an injection current of 30 mA was applied to the VCSELs for a period of 60 s and finally the maximum and minimum surface temperature of the devices was determined for both heating (current applied into the device) and cooling (without current applied into the device) operation times, respectively.

**Fig. 4:**
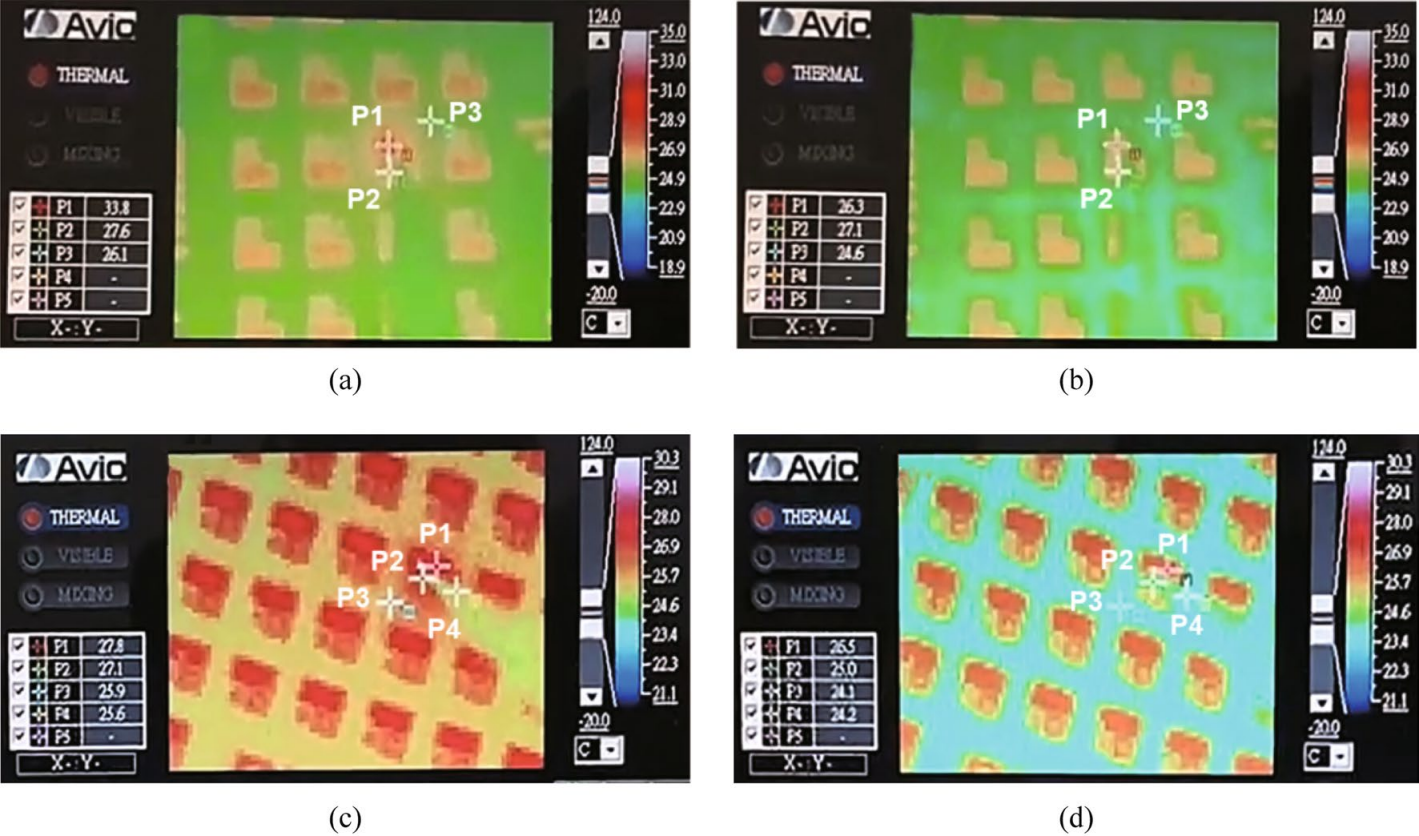
Distribution of temperature of the VCSEL device surface with **a** GaAs substrate during heating; **b** GaAs substrate during cooling; **c** CIC substrate during heating; and **d** CIC substrate during cooling

Figure 4 shows the temperature distribution across the surface of VCSEL/GaAs and VCSEL/CIC during heating and cooling times. The VCSEL/GaAs and VCSEL/CIC had maximum temperatures of 33.8 °C and 27.8 °C, respectively, as shown in Fig. 4a, c and minimum temperatures of 26.3 °C and 26.5 °C, respectively, as presented in Fig. 4b, d. The above results suggested that the thermal management ability of VCSEL/CIC was superior as compared with that of VCSEL/GaAs, owing to the CIC substrate with high thermal conductivity. Moreover, the thinner thickness of CIC substrate also has an impact in the faster heat dissipation by reducing the thermal resistance between the material layers.

### Thermal management capability of VCSEL/GaAs and VCSEL/CIC devices

**Fig. 5:**
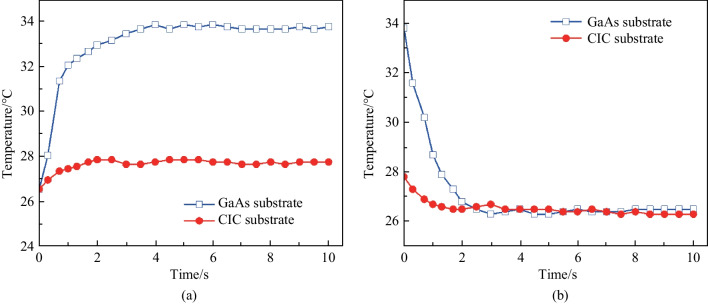
Temperature variation of the VCSEL/GaAs and VCSEL/CIC devices during **a** heating operation time; and **b** cooling operation time

To assess the thermal-transfer ability of the VCSELs with GaAs and CIC substrates, the surface temperature variations as function of time during heating and cooling of the VCSEL devices were recorded simultaneously when performing the measurements of the surface temperature distribution throughout the VCSELs [51–54], as illustrated in Fig. 5. The VCSEL devices in this study were evaluated using the following procedure. The temperature–time variation during heating process, all samples were maintained at 25 °C of atmospheric temperature for 60 s to ensure that the experimental conditions were even and later an injection current of 30 mA was applied to heat the samples; for the temperature–time variation during cooling process, all specimens were kept with an injection current of 30 mA for 60 s to maintain the VCSEL devices at their maximum operating temperature and thereafter the aforementioned current was turned off to enable the samples to cool down.

According to the results shown in Fig. 5a, both VCSEL/GaAs and VCSEL/CIC presented a very fast thermal conduction ability from the starting point of the test. For example, after heating the samples for 2 s, the temperature of the VCSEL/CIC was approximately 27.8 °C and almost maintained constant, which is significantly lower than that of the VCSEL/GaAs (32.9 °C @ 2 s). In contrast, the temperature of the VCSEL/GaAs increased from 26.5 °C to saturated temperature 33.8 °C took about 4 s. The saturation temperature (27.8 °C) of VCSEL with CIC substrate was obviously lower than that (33.8 °C) of VCSEL with GaAs substrates. Furthermore, after cooling the specimens for 2 s, the temperature of the VCSEL/CIC reached around (26.5 °C), which is slightly lower than that of the VCSEL/GaAs (26.8 °C), as shown in Fig. 5b. Moreover, although both VCSEL/GaAs and VCSEL/CIC exhibited very similar surface temperature during the cooling operation, it is noteworthy that their heat dissipation time was quite different; for instance, the VCSEL/CIC reached the surface temperature (26.5 °C) in approximately 1.5 s, while the VCSEL/GaAs presented the aforementioned temperature in approximately 2.7 s. These results indicate that the VCSEL/CIC device shows a much larger heat sinking potential as compared with that of VCSEL/GaAs, which was attributed to the higher thermal conductivity, thermal diffusivity and heat capacity of the CIC composite metal substrate. The obtained surface temperature behaviors of VCSEL/CIC and VCSEL/GaAs were consistent with the thermal characteristics discussed in Fig. 2. As a result, it derived a more rapid thermal-transfer response. As observed by the outstanding thermal management capabilities, the VCSEL/CIC possess remarkable heat transfer ability, making them promising next-generation VCSELs with high-power and high-efficiency that are effective at temperature variations throughout device operation.

**Fig. 6:**
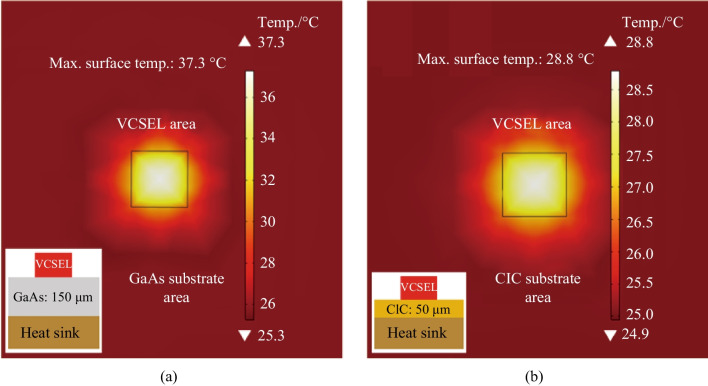
COMSOL simulation results for thermal dissipation from the specimens at steady state for **a** VCSEL/GaAs; and **b** VCSEL/CIC devices

In addition, COMSOL software was employed for further understanding and comparation with the experimental results obtained in this study [1], as illustrated in the Fig. 6. An atmospheric temperature of 25 °C was utilized. The VCSEL device was taken into account as a heat source where released heat energy at a rate of 2.4 × 10^8^ W/m^2^ generated by the power dissipated in the laser with an aperture area of 8 μm, later heat conducted to GaAs substrate with thickness of 150 μm and surface area of 200 μm × 200 μm. As observed in Fig. 6a, b, maximum surface temperatures of 37.3 °C and 28.8 °C were obtained for the VCSEL/GaAs and VCSEL/CIC, respectively. The aforementioned results indicates that the heat dissipation of VCSEL/CIC was superior than that of VCSEL/GaAs, as a result of the CIC substrate with high thermal conductivity, additionally, it was observed that the simulation results were consistent with the experimental results shown in Fig. 4.

## Conclusion

The p-side up VCSEL epilayers with 940 nm wavelength and 150-μm-thick GaAs substrate can be successfully transferred to CIC substrate without cracking by double wafer bonding technology. It was attributed to the CTE of CIC being matched to that of GaAs substrate. Based on the obtained results, the VCSEL with 50-μm-thick CIC substrate exhibited improved optoelectronic properties and the wavelength variation was less than that of VCSEL/GaAs. They were ascribed that the inherent thermal properties of the CIC substrate provided a more rapid heat-conduction response and also to the thinner thickness, which contributes to reducing the thermal resistance from the device to the heat sink.

## Data Availability

All data are available upon request from the corresponding author.

## References

[1] Pan PC, Nag D, Khan Z, Chen CJ, Shi JW, Laha A, Horng RH (2020). Effect of thermal management on the performance of VCSELs. IEEE Trans. Electron Devices.

[2] Grabherr M, Moench H, Pruijmboom A (2013). VCSELs. Springer Ser. Opt. Sci..

[3] Mateus CFR, Huang CJ, Chang-Hasnain CJ, Foley JE, Beatty R, Li P, Cunningham BT (2004). Ultra-sensitive immunoassay using VCSEL detection system. Electron. Lett..

[4] Mateus CFR, Huang MC, Foley J, Beatty PR, Li P, Cunningham BT, Chang-Hasnain CJ (2004). Ultracompact high-sensitivity label-free biosensor using VCSEL. Proc. SPIE.

[5] Plant DV, Trezza JA, Venditti MB, Laprise E, Faucher J, Razavi K, Chateauneuf M, Kirk AG, Luo W (2000). 256-channel bidirectional optical interconnect using VCSELs and photodiodes on CMOS. Proc. SPIE.

[6] Mukoyama N, Otoma H, Sakurai J, Ueki N, Nakayama H (2008). VCSEL array-based light exposure system for laser printing. Proc. SPIEE.

[7] Westbergh P, Gustavsson JS, Haglund Å, Skold M, Joel A, Larsson A (2009). High-speed, low-current-density 850 nm VCSELs. IEEE J. Sel. Top. Quantum Electron..

[8] Al-Omari AN, Alias MS, Ababneh A, Lear KL (2012). Improved performance of top-emitting oxide-confined polyimide-planarized 980 nm VCSELs with copper-plated heat sinks. J. Phys. D Appl. Phys..

[9] Shih TT, Chi YC, Wang RN, Wu CH, Huang JJ, Jou JJ, Lee TC, Kuo HC, Lin GR, Cheng WH (2017). Efficient heat dissipation of uncooled 400-Gbps (16 × 25-Gbps) optical transceiver employing multimode VCSEL and PD arrays. Sci. Rep..

[10] Robertson J, Hejda M, Bueno J, Hurtado A (2020). Ultrafast optical integration and pattern classification for neuromorphic photonics based on spiking VCSEL neurons. Sci. Rep..

[11] Sheng X, Robert C, Wang S, Pakeltis G, Corbett B, Rogers JA (2015). Transfer printing of fully formed thin-film microscale GaAs lasers on silicon with a thermally conductive interface material. Laser Photonics Rev..

[12] Vega-Flick A, Jung D, Yue S, Bowers JE, Liao B (2019). Reduced thermal conductivity of epitaxial GaAs on Si due to symmetry-breaking biaxial strain. Phys. Rev. Mater..

[13] Nadri S, Moore CM, Sauber ND, Xie L, Cyberey ME, Gaskins JT, Lichtenberger AW, Barker NS, Hopkins PE, Zebarjadi M, Weikle RM (2018). Thermal characterization of quasi-vertical GaAs Schottky diodes integrated on silicon. IEEE Trans. Electron Devices.

[14] Nie Q, Chen G, Wang B, Yang L, Tang W (2021). Process optimization, microstructures and mechanical/thermal properties of Cu/Invar bi-metal matrix composites fabricated by spark plasma sintering. Trans. Nonferrous Met. Soc. China.

[15] Jha, S.: CUVAR-a new controlled expansion, high conductivity material for electronic thermal management. IEEE 45th Electronic Components and Technology Conference, 542–547 (1995).

[16] Nie Q, Wang B, Zhang J, Tang W (2022). Fabrication of the Ag-coated Invar/Cu bimetal matrix composites through spark plasma sintering: An investigation on microstructure and properties. Mater. Lett..

[17] Nie QQ, Chen GH, Wang B, Yang L, Zhang JH, Tang WM (2022). Effect of Invar particle size on microstructures and properties of the Cu/Invar bi-metal matrix composites fabricated by SPS. J. Alloys Compd..

[18] Zweben C (1998). Advances in composite materials for thermal management in electronic packaging. Jom.

[19] Kuchta DM, Rylyakov AV, Schow CL, Proesel JE, Baks CW, Westbergh P, Gustavsson JS, Larsson A (2015). A 50 Gb/s NRZ modulated 850 nm VCSEL transmitter operating error free to 90 C. J. Lightw. Technol..

[20] Ledentsov N, Agustin M, Shchukin VA, Kropp JR, Ledentsov NN, Chorchos Ł, Turkiewicz JP, Khan Z, Cheng CL, Shi JW, Cherkashin N (2019). Quantum dot 850 nm VCSELs with extreme high temperature stability operating at bit rates up to 25 Gbit/s at 150 °C. Solid State Electron..

[21] Cheng CL, Ledentsov N, Khan Z, Yen JL, Ledentsov NN, Shi JW (2019). Ultrafast Zn-diffusion and oxide-relief 940 nm vertical-cavity surface-emitting lasers under high-temperature operation. IEEE J. Sel. Topics Quantum Electron..

[22] Moon S, Yun Y, Lee M, Kim D, Choi W, Park JY, Lee J (2022). Top-emitting 940-nm thin-film VCSELs transferred onto aluminum heatsinks. Sci. Rep..

[23] Ouchi T, Sato T, Sakata H (2001). Thin-film vertical-cavity surface-emitting lasers containing strained InGaAs quantum wells fabricated by substrate removal. Jpn. J. Appl. Phys..

[24] Weigl B, Grabherr M, Jung C, Jager R, Reiner G, Michalzik R, Sowada D, Ebeling KJ (1997). High-performance oxide-confined GaAs VCSELs. IEEE J. Sel. Top. Quantum Electron..

[25] Osinski M, Nakwaski W (1995). Thermal analysis of closely-packed two-dimensional etched-well surface-emitting laser arrays. IEEE J. Sel. Top. Quantum Electron..

[26] Choi JH, Wang L, Bi H, Chen RT (2006). Effects of thermal-via structures on thin-film VCSELs for fully embedded board-leveloptical interconnection system. IEEE J. Sel. Top. Quantum Electron..

[27] Hatakeyama H, Anan T, Akagawa T, Fukatsu K, Suzuki N, Tokutome K, Tsuji M (2010). Highly reliable high-speed 1.1-μm-range VCSELs with InGaAs/GaAsP-MQWs. IEEE J. Quantum Electron..

[28] Choi C, Lin L, Liu Y, Chen RT (2003). Performance analysis of 10-μm-thick VCSEL array in fully embedded board level guided-wave optoelectronic interconnects. J. Light. Technol..

[29] Al-Omari AN, Lear KL (2005). VCSELs with a self-aligned contact and copper-plated heatsink. IEEE Photonic Technol. Lett..

[30] Al-Omari AN, Carey GP, Hallstein S, Watson JP, Dang G, Lear KL (2006). Low thermal resistance high-speed top-emitting 980-nm VCSELs. IEEE Photonics Technol. Lett..

[31] Mathine DL, Nejad H, Allee DR, Droopad R, Maracas GN (1996). Reduction of the thermal impedance of vertical-cavity surface-emitting lasers after integration with copper substrates. Appl. Phys. Lett..

[32] Tian SC, Ahamed M, Larisch G, Bimberg D (2022). Novel energy-efficient designs of vertical-cavity surface emitting lasers for the next generations of photonic systems. Jpn. J. Appl. Phys.. J. Appl. Phys..

[33] Tian SC, Mansoor A, Bimberg D (2023). Progress in energy-efficient high-speed vertical-cavity surface-emitting lasers for data communication. MDPI Photonics.

[34] Horng RH, Sinha S, Lee CP, Feng HA, Chung CY, Tu CW (2019). Composite metal substrate for thin film AlGaInP LED applications. Opt. Express.

[35] Sinha S, Feng H, Chung C, Tu C, Horng R (2019). Comparison of properties of thin film AlGaInP LEDs with composite metal and Si substrates. ECS J. Solid State Sci. Technol..

[36] Horng, R. H., Sinha, S., Feng, H. A., Chung, C. Y., Tu, C. W.: Novel composite substrates for thin film AlGaInP-based high power LEDs. In: IEEE 2019 Proceedings of Compound Semiconductor Week (CSW), pp 1–1 (2019)

[37] Horng RH, Wuu DS, Wei SC, Tseng CY, Huang MF, Chang KH, Liu PH, Lin KC (1999). AlGaInP light-emitting diodes with mirror substrates fabricated by wafer bonding. Appl. Phys. Lett..

[38] Sinha S, Tarntair F, Ho C, Wu Y, Horng RH (2021). Investigation of electrical and optical properties of AlGaInP red vertical micro-light-emitting diodes with Cu/Invar/Cu metal substrates. IEEE Trans. Electron Devices.

[39] Perera IU, Narendran N, Liu Y (2013). Accurate measurement of LED lens surface temperature. Proc. SPIE.

[40] Dai S, Li J, Lu N (2020). Research progress of diamond/copper composites with high thermal conductivity. Diam. Relat. Mater.Relat. Mater..

[41] Lee SH, Kwon SY, Ham HJ (2012). Thermal conductivity of tungsten–copper composites. Thermochim. Acta.

[42] Deppe DG, Li M, Yang X, Bayat M (2018). Advanced VCSEL technology: self-heating and intrinsic modulation response. IEEE J. Quantum Electron..

[43] Mogg S, Chitica N, Christiansson U, Schatz R, Sundgren P, Asplund C, Hammar M (2004). Temperature sensitivity of the threshold current of long-wavelength InGaAs-GaAs VCSELs with large gain-cavity detuning. IEEE J. Quantum Electron..

[44] Filipchuk A, Nechay K, Ulkuniemi R, Talmila S, Uusimaa P (2022). Thermal management optimization in high-power 3D sensing VCSELs. Proc. SPIE.

[45] Haglund EP, Kumari S, Haglund E, Gustavsson JS, Baets RG, Roelkens G, Larsson A (2016). Silicon-integrated hybrid-cavity 850-nm VCSELs by adhesive bonding: impact of bonding interface thickness on laser performance. IEEE J. Sel. Top. Quantum Electron..

[46] Fedorov SA, Beccari A, Arabmoheghi A, Wilson DJ, Engelsen NJ, Kippenberg TJ (2020). Thermal intermodulation noise in cavity-based measurements. Optica.

[47] Huang SY, Horng RH, Shi JW, Kuo HC, Wuu DS (2009). High-performance InGaN-based green resonant-cavity light-emitting diodes for plastic optical fiber applications. J. Lightwave Technol..

[48] Huang SY, Horng RH, Liu PL, Wu JY, Wu HW, Wuu DS (2008). Thermal stability improvement of vertical conducting green resonant-cavity light-emitting diodes on copper substrates. IEEE Photonics Technol. Lett..

[49] Cho J, Sone C, Park Y, Yoon E (2005). Measuring the junction temperature of III-nitride light emitting diodes using electro-luminescence shift. Phys. Status Solidi A.

[50] Gu, Y., Narendran, N.: A noncontact method for determining junction temperature of phosphor-converted white LEDs. In: Third international conference on solid state lighting, Proc. SPIE **5187**, 107–114 (2004).

[51] Chen X, Lim JSK, Yan W, Guo F, Liang YN, Chen H, Lambourne A, Hu X (2020). Salt template assisted BN scaffold fabrication toward highly thermally conductive epoxy composites. ACS Appl. Mater. Interfaces.

[52] Liu L, Xiang D, Wu L (2022). Improved thermal conductivity of ceramic-epoxy composites by constructing vertically aligned nanoflower-like AlN network. Ceram. Int..

[53] Lee Sanchez WA, Li JW, Chiu HT, Cheng CC, Chiou KC, Lee TM, Chiu CW (2022). Highly thermally conductive epoxy composites with AlN/BN hybrid filler as underfill encapsulation material for electronic packaging. Polymers.

[54] Yan R, Su F, Zhang L, Li C (2019). Highly enhanced thermal conductivity of epoxy composites by constructing dense thermal conductive network with combination of alumina and carbon nanotubes. Compos. Part A Appl. Sci..

